# Women’s Challenges and Capabilities in Disasters: A Case Report of the Twin Earthquakes of Eastern Azerbaijan, Iran

**DOI:** 10.1371/currents.dis.2cff3d6e9e0c3a597f873bf29e712370

**Published:** 2017-02-22

**Authors:** Sanaz Sohrabizadeh, Katayoun Jahangiri, Reza Khani Jazani, Javad Babaie, Mohammad Javad Moradian, Behnaz Rastegarfar

**Affiliations:** Department of Health in Disasters and Emergencies, School of Health, Safety and Environment, Shahid Beheshti University of Medical Sciences, Tehran, Iran; Associate Professor in School of Health, Safety and EnvironmentShahid Beheshti University of Medical Sciences; Shahid Beheshti University of Medical Sciences; Department of Health Services Management, School of Management and Medical Informatics, Tabriz University of Medical Sciences, Tabriz, Iran; Iranian Center of Excellence in Health Management, School of Management and Medical Informatics, Tabriz University of Medical Sciences, Tabriz, Iran; Department of Disaster Public Health, School of Public Health, Tehran University of Medical Sciences, Tehran, Iran; Trauma Research Center, Shahid Rajaee (Emtiaz) Trauma Hospital, Shiraz University of Medical Sciences, Shiraz, Iran; Dept. of Disaster and Emergency Health, I.R.Iran’s National Institute of Health Research, Tehran University of Medical Sciencesnational institute of health research

## Abstract

**Introduction**: The twin earthquakes of eastern Azerbaijan induced considerable devastations of many villages and cities. About 70% of all victims were women and children. The present case report was aimed at describing the lessons learnt from both capabilities and challenges of girls and women after the twin earthquakes of eastern Azerbaijan.

**Methods**: A qualitative approach using in-depth unstructured interviews was used for this study. A total of 13 participants (two men and 11 women), affected by the quakes, were interviewed applying the purposeful sampling method. A manifest content analysis was performed for analyzing the transcribed interviews

**Case presentation**: Two categories of women’s capabilities and challenges and four subcategories of women’s participation in community reconstruction, livelihood efforts, aggravated poverty and violence were extracted from the data which were gathered in the destroyed fields of eastern Azerbaijan.

**Lessons learnt**: Women can play an important role in post-disaster recovery. Consequently, ignoring the challenges and capabilities of women may impede post-disaster development processes, which would adversely affect the whole community.

## Introduction

Gender is considered as one of the key aspects of disaster vulnerability and it is the main aspect of social discrimination.^1 ^ Iran is known to be a highly disaster-prone country^2^ with an almost equal gender distribution (49.6% for women).^3^ During the last three decades, Iranian people have suffered from the destructive effects of natural disasters including earthquakes, floods, and droughts.^2^ Women and girls have been affected by negative consequences of natural disasters more than men. For example, **some women **were the victims of rapes and sexual harassment after the Bam earthquake in 2003**.** 4 In addition, a number of studies reported several mental health disorders among the affected women included depression, anxiety and post-traumatic stress disorders (PTSD).^5^^,^ 6 Furthermore, joblessness, indigence, violence, and psychosomatic diseases have been reported as women’s challenges in some disaster-stricken regions in the world.^7^^,^ 8 ^,^ 9 ^,^ 10

The twin earthquakes of eastern Azerbaijan province in 2012 (12 August) caused considerable damage in many villages and cities. Furthermore, the earthquakes killed more than 300 people and injured about 3000 citizens living in the affected regions. About 70% of all killed people were women and their children.^11^ The present case report was aimed at identifying the capabilities and challenges of women after the twin earthquakes of eastern Azerbaijan. This report was also meant to summarize the lessons learnt from the women’s experiences after the twin earthquakes of eastern Azerbaijan.

**Methods**

A qualitative approach using content analysis was applied for the study. Affected people living in the destroyed regions of eastern Azerbaijan were approached for interviews. A purposeful sampling method was used for selecting participants in the disaster-affected fields. Native health officials working in the public health centers of the damaged regions helped the researchers by preparing a list of affected people. The list included addresses and contact information of damaged households.

The number of participants was determined based on the saturation principles. Data saturation was reached after 12 interviews, but one additional interview was conducted to make certain that no new concept emerged. A total of 13 participants (two men and 11 women) were interviewed. Data were collected through in-depth unstructured interviews carried out in the destructed houses and conex (or temporary containers). The first author asked each respondent “tell me about your post-disaster experiences.” Probing was performed to encourage the participants to describe their experiences and feelings completely. Each interview was analyzed immediately, and the retrieved data became a guide for further data collection.

Data gathering and data analysis were performed simultaneously. Several steps were conducted for data analysis. First, the interviews were read several times to obtain a sense of the whole. Second, the entire material was brought together into a single text which formed the unit of the analysis. Third, the text was divided into meaningful units. Fourth, the condensed meaningful units were labeled with a code. Finally, the codes were compared according to the differences and similarities and sorted into two categories and four subcategories.

Member and expert checking as well as sampling triangulation (including men and women) were used for achieving trustworthiness. In member checking, the main researcher asked the respondents about possible misunderstandings during interviews and for expert checking, the research team discussed the emerging subjects.

**Case Presentation**

All participants were in the age range of 17-60 years with educational levels from illiteracy (15%) to diploma education (15%). About 70% of all participants had primary education. The majority were female (77%) and the remaining (23%) were male.

Two categories of women’s challenges and women’s capabilities were extracted from the data: Women's capabilities and Women's challenges. These categories consisted of four subcategories, namely, participation in community reconstruction, livelihood efforts, aggravated poverty and violence.

**1-** **Women’s capabilities**

**1-1-** **Participation in Community Reconstruction: **Reconstruction of the destroyed regions were indirectly initiated by the government’s loans for damaged houses. Households took care of all reconstruction costs through the loan that was given. Since the government’s payment was not enough to take care of the reconstruction costs, women and girls participated in reconstruction of their damaged houses in conjunction with men. Consequently, women helped the men to save money by working as builders. On the other hand, reconstruction was an imperative priority for all the people affected owing to the fact that living in conex and temporary settlements was difficult at the onset of winter.

**1-2-** **Livelihood Efforts: **Carpet weaving was the main source of income in the affected regions and was stopped due to the destruction and inappropriate living conditions in conex or temporary settlements. Carpet weaving was done by women and young girls in newly reconstructed houses. Although some instruments used for weaving were destroyed in the earthquakes, carpet weaving was the main source of livelihood for women and girls after the quakes. In addition, after the quakes. Since livestock were killed and their houses (barns) were destroyed during the earthquakes, farming and ranching activities were not possible and carpet weaving was the only source of income in the affected regions.

**2-** **Women’s challenges**

**2-1-** **Aggravated Poverty: **Pre- and post-disaster poverty as well as economic burden were reported in the affected regions. Consequently, poverty exacerbated in the damaged regions after the earthquakes. Women and girls were compelled to stay at home and weave carpet and engage in farming as a domestic job. However, shrinking of carpet and inadequate space for weaving were the challenges of post-disaster carpet weaving, which exposed them to the risk of further impoverishment. In addition, some young girls stopped going to high school and could not obtain their diploma because their family could not afford the cost of their education after the earthquakes.

**2-2-** **Violence: **Early and forced marriages were forms of violence observed against women and girls before and after disasters; however, forcing****teenage girls into marriage was common after the earthquakes due to post-disaster poverty. Furthermore, forcing of young girls to work at home and banning them from having access to education were considered as other forms of violence against women in the affected regions. Women and girls had to be allowed by men (father, husband, brother, and uncle) to leave home for work, attend school or even receive relief aids after the earthquakes. For instance, a young woman who had diploma in information technology stated that her husband did not allow her to work with computers, but allowed her to weave carpet and work in the farm.

## Lessons Learnt

According to the present case report, women suffered from poverty and violence after the twin earthquakes of eastern Azerbaijan. On the contrary, the affected communities benefit from the women’s capabilities included participation in community reconstruction and livelihood efforts. In consistence with the report, affected women faced joblessness, poverty and domestic violence after the Sri Lanka tsunami.^9^ Furthermore, the report of disaster-affected regions in India revealed that women suffered from early marriage, poverty, lack of education and violence after the quakes.^10^ The following lessons can be learnt from the results of this case report:

- Women were confronted with the effects of poverty and violence before the earthquakes and these challenges were exacerbated after the twin earthquakes. Disasters provide a unique opportunity for development and it is highly recommended that the women’s challenges be assessed after disasters with a view to improving their quality of lives as well as initiating positive changes after disasters.
- Women and girls assisted men with reconstruction of the affected regions. Women can be the primary actors in community based disaster management; however, sociocultural factors should be taken into consideration in post-disaster recovery and development measures.
  - Knowledgeable women can play key roles in achieving a developed and resilient community. Preventing women from having access to education and reducing their social interaction can exacerbate their poverty and make them more susceptible to future disasters.
- Domestic jobs done by women and girls were the main source of income after the earthquakes. Furthermore, planning and engaging in entrepreneurship projects in the affected regions can reduce economic challenges and improve the welfare status of the whole community during the post-disaster phase.
- Although women played an important role in providing family livelihood, however, their tasks and workload increased after the earthquakes. Ignoring women’s health and well-being status can adversely influence children, households and the entire community.
- A sex disaggregated database has not been developed yet and gender analysis was not performed in previous disasters in Iran. Gender analysis tools and indicators for assessing post-disaster status of affected women should be developed within the context of disaster in Iran.
- Women had little information on their fundamental rights and considered behaviors related to violation of their rights as normal and inevitable. Violence against women and girls can be mitigated through a context based planning and management that involves men’s participation.
- Regarding the fourth priority of the Sendai Framework for Disaster Risk Reduction, gender equality should be promoted in reconstruction and rehabilitation plans within the context of disaster in Iran. Empowerment of women and girls can reduce their susceptibilities and expedite post-disaster development.

## Ethical Consideration

This study was approved by the ethics committee of Shahid Beheshti University of Medical Sciences (Tehran, Iran). All participants were informed about confidentially of their names and other private information in the related reports.

## Corresponding Author

Katayoun Jahangiri (k.jahangiri@sbmu.ac.ir)

## Competing Interests

The authors have declared that no competing interests exist.

## Data Availability

The data set contains interview transcripts and will only be shared upon request. For further information regarding data availability please contact Sanaz Sohrabizadeh at sohrabizadeh@sbmu.ac.ir
